# Curcumin Modulates TIGIT/Neuropilin-1 to Regulate T-Cell Immune Homeostasis in Ulcerative Colitis

**DOI:** 10.3390/foods14244323

**Published:** 2025-12-15

**Authors:** Yazhen Liu, Jiaqi Huang, Ji Yu, Luxin Fu, Ronglong Huang, Jing Liu, Bailin Deng, You-Bao Zhong, Duanyong Liu, Haimei Zhao

**Affiliations:** 1 Graduate School, Jiangxi University of Chinese Medicine, Nanchang 330004, China; liuyazhen@jxutcm.edu.cn (Y.L.); huangjiaqi46@jxutcm.edu.cn (J.H.); yuji1@jxutcm.edu.cn (J.Y.); fuluxin@jxutcm.edu.cn (L.F.); huangronglong@jxutcm.edu.cn (R.H.); liujing42@jxutcm.edu.cn (J.L.); 2 Formula-Pattern Research Center, Jiangxi University of Chinese Medicine, Nanchang 330004, China; 20211050@jxutcm.edu.cn (B.D.); zhong-youbao@foxmail.com (Y.-B.Z.); 3 Key Laboratory of Prevention and Treatment of Immunological and Metabolic Diseases Related to Prescription and Syndrome, Nanchang 330004, China; 4 School of Nursing, Jiangxi University of Chinese Medicine, Nanchang 330004, China

**Keywords:** curcumin, ulcerative colitis, TIGIT, Neuropilin-1, CD4^+^ T cells, memory T cells

## Abstract

(1) Background: Ulcerative colitis (UC) is a persistent inflammatory condition of the intestine, characterized by dysregulated T cell-mediated immune responses. Curcumin (CUR), a common food additive and health supplement, is noted for possessing anti-inflammatory and immunomodulatory properties. Nevertheless, the molecular mechanisms underlying its therapeutic effects remain incompletely elucidated. This research aims to investigate the therapeutic mechanisms of CUR in UC, focusing on its role in restoring T cell homeostasis by modulating TIGIT and Neuropilin-1 (NRP1). (2) Methods: We employed a DSS-induced murine colitis model, combined with network pharmacology, molecular docking, protein–protein interaction docking, molecular dynamics simulations, and *in* *vitro* assays with Jurkat T cells. (3) Results: CUR markedly ameliorated clinical manifestations and histopathology in DSS-treated mice, restoring the balance of T cell and memory T cell subsets. Computational predictions and experimental validation showed that CUR downregulated TIGIT and NRP1 expression in inflamed colonic tissue and directly inhibited their expression in activated T cells *in* *vitro*. (4) Conclusions: This study reveals a novel immunoregulatory mechanism of this natural compound. These findings suggest CUR modulates TIGIT/NRP1 to inhibit excessive T cell activation and restore immune homeostasis in UC.

## 1. Introduction

Ulcerative colitis (UC) is recognized as a principal clinical subtype of inflammatory bowel disease (IBD). From a clinicopathological standpoint, the hallmark of this condition is chronic, recurrent inflammatory injury to the colonic mucosa, which is accompanied by characteristic ulcerative alterations [1,2,3]. While its exact pathogenesis remains incompletely elucidated, current paradigms generally attribute UC to complex crosstalk between genetic susceptibility, environmental triggers, and aberrant immune responses [4,5]. Notably, T cell-mediated immune dysregulation constitutes a primary driver in the pathophysiological process of UC [6]. This manifests as excessive activation of pro-inflammatory T-cell subsets, including Th1 and Th17 cells, alongside a diminished function of regulatory T cells (Tregs) [7]. This imbalance leads to sustained high expression of pro-inflammatory cytokines (such as IL-6, IL-17A, TNF-α) alongside a marked reduction in anti-inflammatory cytokines, including TGF-β1, IL-10, ultimately causing inflammatory damage and functional impairment of the intestinal mucosa [8]. Accordingly, developing therapeutic strategies that can restore T cell immune homeostasis and effectively suppress inflammatory responses is crucial for clinical management in UC.

Curcumin (CUR), a naturally occurring polyphenolic compound extracted from Curcuma longa, has garnered substantial attention owing to its notable anti-inflammatory, antioxidant, and immunomodulatory properties [9]. Clinical cases have demonstrated the potential of CUR in treating ulcerative colitis [10]. Its multifaceted mechanism of action has been elucidated to involve the inhibition of key inflammatory signaling pathways, thereby downregulating the secretion of pro-inflammatory cytokines [11,12,13]. Although the broad-spectrum anti-inflammatory properties of CUR are well-documented, the precise mechanisms by which it modulates T cell subset balance to achieve therapeutic effects in UC remain poorly understood and warrant further comprehensive preclinical pharmacological exploration. In recent years, T-cell immunoreceptor with Ig and ITIM domains (TIGIT) and Neuropilin-1 (NRP1) have been recognized as pivotal regulators governing T lymphocyte activation and function [14].

As a newly discovered immunosuppressive receptor, TIGIT exerts its inhibitory effects through two primary mechanisms: (1) phosphorylation of its intracellular tyrosine inhibitory motif (ITIM) recruits signaling molecules to suppress downstream pathways, directly diminishing T cell activity [15]; (2) TIGIT binds its ligand CD155 with higher affinity than the activating receptor CD226, thereby competitively blocking co-stimulatory signals and ultimately promoting immune tolerance and T cell exhaustion [16]. The function of NRP1 is more complex. This factor is markedly upregulated in activated effector T cells. Its increased expression is directly associated with the pathological progression observed in various autoimmune diseases, thereby suggesting a critical pro-disease role in inflammatory pathogenesis [17]. In patients with IBD, the percentage of NRP1-positive Treg cells in the intestinal mucosa and peripheral blood is typically elevated [18], potentially reflecting the body’s attempt at immune regulation to compensate for and suppress persistent inflammation. Given the critical roles of TIGIT and NRP1 in precisely regulating T cell activation, differentiation, and immune tolerance, they have emerged as potential therapeutic targets for developing treatment strategies targeting UC.

Although CUR shows significant therapeutic promise in various inflammatory and immune-mediated disorders, no direct evidence in the existing literature supports its ability to regulate TIGIT and NRP1. Given CUR’s well-documented potent immunomodulatory efficacy in UC treatment and the pivotal function of TIGIT/NRP1 in T cell activation, differentiation, and maintenance of immune tolerance, we propose that CUR may mediate its therapeutic effects through modulating TIGIT/NRP1 to regulate T-Cell immune homeostasis. To systematically elucidate how CUR modulates TIGIT/NRP1 and thereby restores T-cell immune homeostasis to alleviate UC, this study will integrate multidisciplinary approaches. This study will validate a DSS-induced mouse model and use computational biology methods to predict interactions between CUR and immune checkpoint molecules TIGIT and NRP1. Furthermore, *in* *vitro* cellular functional validation experiments will confirm CUR’s direct effects on T cell activity, TIGIT/NRP1 expression, and other cytokines.

## 2. Materials and Methods

### 2.1. Pharmaceutical Agents and Chemical Reagents

Dextran sulfate sodium (Molecular mass: 36–50 kDa; Lot: 160110) was procured from MP Biomedicals (Irvine, CA, USA). Curcumin (Lot: HY-N0005) and Phorbol 12-myristate 13-acetate (PMA, Lot: HY-18739) were purchased from MedChemExpress (MCE, NJ, USA). Carboxymethylcellulose Sodium (CMC-Na, Lot: C8621) and Ionomycin Calcium Salt (Iono, Lot: I8800) were procured from Solarbio (Beijing, China). Tiragolumab (anti-TIGIT) (Lot: Ab169250) and Vesencumab (anti-NRP1) (Lot: Ab183435) were obtained from Aladdin (Shanghai, China). Fecal OB-COMPLETE (Lot: BA2020E) was acquired from Zhuhai Baso Biotechnology (Zhuhai, China). ELISA kits (such as IL-10, TGF-β1, IL-6, IL-17A, and TNF-α) were acquired from Thermo Fisher (Vienna, Europe). The real-time PCR kits-Rapid Tissue/Cell RNA Extraction Kit (Lot: RN68), TRUEscript RT MasterMix (OneStep gDNA Removal) Reverse Transcription Kit (Lot: PC7001), and 2× Dual Sybr Green qPCR Mix (Universal ROX) Reagent (Lot: PC6201) were sourced from Aidlab (Beijing, China). Flow cytometry antibodies: AF647 Rat Anti-mouse CCR7, V450 Rat Anti-mouse CCR6, V500 and APC Rat Anti-mouse CD4, PE Rat Anti-mouse CXCR3, PE-Cy7 Rat Anti-mouse IFN-γ, PE Rat Anti-mouse IL-17A, V450 and PE Rat Anti-mouse Foxp3 were provided by BD Biosciences(Franklin, CA, USA). Specific antibodies for Western blotting and immunofluorescence (TIGIT, NRP1) were obtained from ProteinTech Group, Inc. (Wuhan, China).

### 2.2. Mice

Male specific pathogen-free (SPF) C57BL/6 mice, aged 7–8 weeks and weighing 22 ± 2 g, were obtained from GP (Nanjing, China) [Animal Certificate number: SYXK(Su)2023-0009]. All animal procedures were performed in strict compliance with the protocols established by the Institutional Animal Care and Use Committee at the Animal Facility of Jiangxi University of Chinese Medicine (Nanchang, China) [Animal Use Permit number: SYXK(Gan)2022-0002]. Forty mice were housed under standard conditions, permitted to breed in a natural manner, and given ad libitum access to standard laboratory feed and water. This study protocol (Identification number: JZLLSC20230721) received approval from the Institutional Animal Care and Use Committee of Jiangxi University of Chinese Medicine.

### 2.3. Induction of Colitis Model

**Figure 1:**
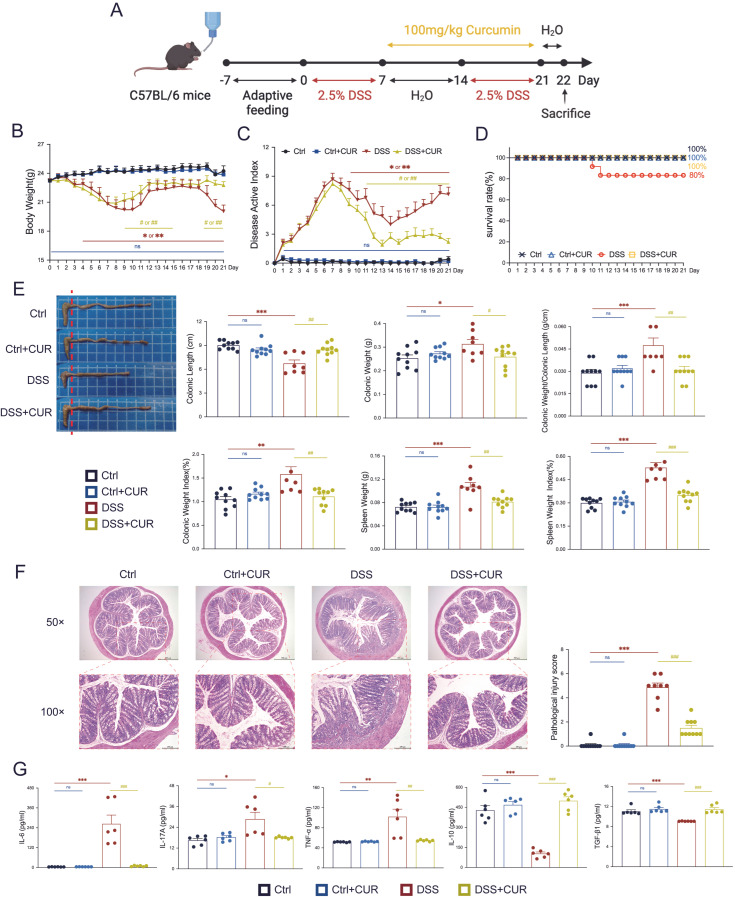
CUR effectively alleviates DSS-induced UC in mice. (**A**) Replicating the experimental model of colitis and administering CUR. (**B**) Body weight changes were observed across the four experimental murine cohorts over the 21-day study period. (**C**) The DAI scores were recorded across the four treatment cohorts throughout the 21-day experimental period. (**D**) Survival rates in the four groups of mice. (**E**) Colon length, Colon weight, Colon weight/Colon Length, Colon weight index, spleen weight, and spleen weight index on day 22 for four groups of mice. The red dashed line indicates the starting point of the colon measurement. (**F**) Representative micrographs illustrating the histopathology of murine colonic sections from the distinct experimental cohorts (HE staining). Images are presented at two magnifications: 50× (scale bar = 500 μm) and 100× (scale bar = 200 μm). (**G**) Quantitative analyses of pro-inflammatory mediators (IL-6, IL-17A, TNF-α) and regulatory mediators (IL-10, TGF-β1) were performed on colonic tissues from various experimental groups. The data are expressed as the mean ± SEM (*n* = 6–10). Significantly different (* p < 0.05, ** p < 0.01, *** p < 0.001) vs. the Ctrl group. Significantly different (*^#^ p* < 0.05, *^##^ p* < 0.01, *^###^ p* < 0.001) vs. the DSS group.

Following a 7-day period of adaptive feeding, the mice commenced unrestricted consumption of 2.5% (*w*/*v*) DSS (Figure 1A). This was succeeded by a 7-day interval of regular water consumption, after which the 2.5% (*w*/*v*) DSS treatment was reintroduced for an additional 7 days. The first day of administering the 2.5% (*w*/*v*) DSS solution was designated as day 0 of the experiment. After successfully inducing the ulcerative colitis model with DSS, mice typically present with clinical manifestations including weight loss, diarrhea or loose stools, and bloody stool. In the subsequent experimental procedure, forty C57BL/6 mice were randomly allocated to 4 groups, with each group consisting of ten individuals (*n* = 10): a blank control group (Ctrl), a CUR control group (Ctrl + CUR), a DSS model group (DSS), and a CUR treatment group (DSS + CUR).

### 2.4. CUR Treatment Protocol

To assess the therapeutic efficacy of CUR, it was dissolved in 0.5% Carboxymethylcellulose Sodium. Beginning on day 7, the mice were administered daily gavage treatments in accordance with their respective group assignments. The DSS and Ctrl group received physiological saline, whereas the Ctrl + CUR and DSS + CUR group were administered CUR at a dosage of 100 mg/kg/day (Figure 1A) [19]. Throughout the duration of the study, the body weight of all animals was recorded every day at 10:00 AM. Additionally, symptoms including diarrhea, presence of blood in the stool, hunching, and hair loss were meticulously recorded to evaluate disease severity.

### 2.5. Macroscopic Efficacy Assessment

On day 22, all mice were weighed and euthanized under sodium pentobarbital anesthesia (20 mg/kg, i.p.). The abdomen was then promptly opened, and the spleen and colon tissues were carefully dissected. After flushing the colon contents with physiological saline, we measured the length and weight of the colon. The colon weight index was calculated as: colon weight/mouse weight × 100%. Similarly, following the measurement of spleen weight, we calculated the spleen weight index using the formula: spleen weight/mouse weight × 100%.

### 2.6. Disease Activity Index (DAI)

During the experiment, the body mass of all animals was recorded once daily at a consistent time, and their faecal consistency and occult blood were assessed. DAI was calculated utilizing the following parameters: Weight Loss Rate (No significant loss, 0; 1–5% loss, 1; 6–10% loss, 2; 11–20% loss, 3; >20% loss, 4), degree of stool consistency (Normal stool, 0; Soft but still formed stool, 1; Soft stool, 2; Wet stool, 3; Watery diarrhea stool, 4), and degree of bleeding (Negative hemoccult, 0; Positive hemoccult, 2; Visible blood traces in stool, 3; Severe rectal bleeding, 4) [20]. DAI Score = Weight Loss Rate Score + Fecal Consistency Score + Fecal Occult Blood Score.

### 2.7. Histopathological Evaluation

On day 22, promptly isolate the mouse colon. Fix the proximal colon in a 4% paraformaldehyde solution for 24 h, followed by sequential processing through graded ethanol dehydration, xylene clearing, and paraffin embedding. Subsequently, section the tissue into 4 μm. Following staining of the sections with HE staining, capture images and conduct pathological evaluation using a light microscope (Leica, Wetzlar, Germany). Two independent pathologists conducted a blinded histological evaluation of colonic pathology, focusing primarily on inflammatory cellular infiltration and tissue structural damage. The scoring system for inflammatory cellular infiltration ranged from 0 to 3, with 0 indicating minimal cellularity confined to the lamina propria, and 3 denoting full transmural penetration. For structural pathology, the severity scale similarly ranged from 0, representing intact mucosa, to 3, indicating extensive damage reaching the basal layers of the intestinal structure [20].

### 2.8. Flow Cytometry Analysis

On day 22 of the experiment, mouse spleens were promptly isolated, and single-cell suspensions were prepared on ice. To begin the procedure, red blood cells were removed using a specific lysis buffer. Resuspend the isolated splenic lymphocytes and monocytes in 1× PBS containing 3% fetal bovine serum, adjusting the cell density to 1 × 10^7^ cells/mL. Subsequently, 250 μL of the cell suspension was transferred into flow cytometry tubes, to which BD Pharmingen™ Leukocyte Activation Cocktail with BD GolgiPlug™ (BD, Franklin, USA, Lot: 550583) was added. Cellular stimulation was conducted over 4–6 h at 37 °C in a 5% CO_2_ atmosphere. Following stimulation, Fc receptor blocking reagent (BD, Franklin, USA, Lot: 553141) was added, and the cellular samples were incubated for 10 min at 4 °C in the dark.

Thereafter, cells were fixed and made permeable using either the BD Cytofix/Cytoperm™ Fixation/Permeabilization Kit (BD BiosciencesFranklin, CA, USA, Lot: 54714) or the BD Pharmingen™ Transcription Factor Buffer Set (BD BiosciencesFranklin, CA, USA, Lot: 562574). This was succeeded by a 30 min cold storage period under light-exclusion conditions at 4 °C. Subsequently, the resulting cell suspensions were analysed using a FACSCanto II instrument (BD, Franklin Lakes, USA), according to the predefined flow cytometry staining panel.

Multiple monoclonal antibodies were utilized for staining purposes, including AF647 Rat anti-mouse CCR7, V450 Rat anti-mouse CCR6, V500 and APC Rat anti-mouse CD4, PE Rat anti-mouse CXCR3, PE-Cy7 Rat anti-mouse IFN-γ, PE Rat anti-mouse IL-17A, as well as V450 and PE Rat anti-mouse Foxp3 antibodies, each diluted at a ratio of 1:100. Subpopulation gating boundaries were established using negative controls and isotype controls. Data analysis was conducted utilizing FlowJo software, version 10.8.1 (Corvallis, OR, USA). A rigorous gating protocol was employed to eliminate extraneous particulate matter and cellular aggregates, thereby improving the accuracy of the quantification.

The detailed gating strategy is as follows: total CD4^+^ T cells are identified as CD4^+^; Treg cells as CD4^+^Foxp3^+^; Th17 cells as CD4^+^IL-17A^+^; Th1 cells as CD4^+^IFN-γ^+^; central memory Treg cells as CD4^+^CCR7^+^Foxp3^+^; central memory Th17 cells as CD4^+^CCR7^+^CCR6^+^; central memory Th1 cells as CD4^+^CCR7^+^CXCR3^+^; effector memory Treg cells as CD4^+^CCR7^−^Foxp3^+^; effector memory Th17 cells as CD4^+^CCR7^−^CCR6^+^; effector memory Th1 cells as CD4^+^CCR7^−^CXCR3^+^; total CD4^+^ T cells expressing TIGIT as CD4^+^TIGIT^+^; central memory T cells expressing TIGIT as CD4^+^CCR7^+^TIGIT^+^; effector memory T cells expressing TIGIT as CD4^+^CCR7^−^TIGIT^+^; central memory Treg cells expressing TIGIT as CD4^+^CCR7^+^Foxp3^+^TIGIT^+^; and effector memory Treg cells expressing TIGIT as CD4^+^CCR7^−^Foxp3^+^TIGIT^+^.

### 2.9. Network Pharmacology

This study employed network pharmacology methodologies to elucidate the mechanisms by which CUR exerts therapeutic effects in ulcerative colitis. Initially, the active constituents of traditional Chinese medicine and their corresponding SMILES data were obtained from PubChem. Potential targets of these compounds were predicted using the SwissTargetPrediction and SEA databases. Concurrently, disease-associated targets relevant to ulcerative colitis were collected and integrated from the GeneCards and OMIM databases. The intersection of these two target sets was determined using Venny software 2.1.0 to identify potential key targets.

Subsequently, the VLOOKUP function was employed to screen for core components most strongly associated with the disease, and a “component-target-disease” network was constructed using Cytoscape 3.8.2, followed by topological analysis. A protein–protein interaction (PPI) network was then established using data from the STRING database. It was designated as Homo sapiens, with a confidence threshold set at 0.900, and orphan gene nodes were hidden. Protein interaction relationships were obtained, and the top ten core targets were identified in Cytoscape based on their degree values. In conclusion, enrichment analyses of the core targets were performed utilizing the Bioconductor package for Gene Ontology (GO) and Kyoto Encyclopedia of Genes and Genomes (KEGG) databases. These analyses aimed to elucidate the therapeutic mechanisms of the drug at multiple levels, encompassing molecular functions, biological processes, cellular components, and signaling pathways.

### 2.10. Protein-Protein Docking

We began the study by retrieving the two-dimensional structural coordinates of the low-molecular-weight ligand, CUR, from PubChem. The three-dimensional structure was subsequently generated using ChemOffice and stored with the .mol2 file extension. Concurrently, atomic-resolution receptor models were obtained from the RCSB repository for molecular docking studies. These models underwent essential preprocessing, which involved the removal of water molecules and phosphate ions using PyMOL v. 2.6, before being exported in PDB format.

Molecular docking simulations were conducted using AutoDock Vina software 1.5.6. Before commencing the procedure, both the protein and ligand structures underwent essential processing using AutoDock version 1.5.6, which included the addition of hydrogen atoms and the definition of docking box coordinates. The assessment of the research findings is principally based on the calculated binding energy (BE) values. In accordance with thermodynamic principles, a lower binding energy corresponds to a higher binding affinity. Specifically, a binding energy below −5.0 kcal/mol is regarded as indicative of favourable active binding, whereas a value below −7.0 kcal/mol strongly implies a high binding affinity. Finally, the docking results were visualized using Discovery Studio 2019 and PyMOL 2.6 software, producing two-dimensional and three-dimensional interaction diagrams illustrating the interactions between CUR and key amino acid residues.

### 2.11. Molecular Dynamics Simulation

Molecular dynamics (MD) simulations were conducted using GROMACS 2022 software. The receptor protein was modelled using the amber14sb force field, whereas the binding partner was represented employing the GAFF2 potential function. Parameters were generated using tools such as pdb2gmx and AutoFF. The system preparation proceeded as follows: the complex was first placed in a cubic box filled with TIP3P water molecules, with a 1 nm buffer region surrounding it [21]. Ions were then added using the gmx genion programme to neutralise the system’s charge. The handling of long-range electrostatic forces was conducted using the Particle Mesh Ewald (PME) method, employing a truncation distance of 1 nm. Additionally, the rigidity of covalent connections was maintained by employing the SHAKE algorithm. Integration was performed with the Verlet leapfrog integrator, employing a time step of 1 fs. Prior to a 100 ns NPT ensemble simulation at 310 K, the system underwent a staged energy minimisation process: initially minimising the water molecules while constraining the solute, then minimising the counterions with solute constraints, and finally performing an unconstrained minimisation of the entire system. This process comprised a series of steepest descent optimisations (3000 steps) followed by conjugate gradient optimisations (2000 steps). Post-simulation analyses were carried out using the relevant GROMACS analysis tools to calculate key spatial and kinetic parameters, comprising RMSD, RMSF, Rg, and SASA.

### 2.12. Cell Culture

The Jurkat cell line (ATCC, Clone E6-1, CL-0129) was cultured in Jurkat Clone E6-1 cell-specific medium (Procell, CM-0129) using 25 cm^2^ or 75 cm^2^ cell culture flasks, maintaining a maximum cell density of 2 × 10^6^ cells/mL. The assay commenced by inoculating Jurkat cells at a density of 2 × 10^4^ cells per well, using multi-well culture plates. This initial plating enabled the determination of the half-maximal inhibitory concentration (IC_50_) of CUR, which was subsequently calculated to be 30.42 μM. Based on this result, CUR concentrations of 1.5, 3, and 6 μM were selected for subsequent experiments. Except for the blank control group, all experimental groups were stimulated with 50 ng/mL Phorbol 12-myristate 13-acetate (PMA, MedChemExpress, HY-18739) and 500 ng/mL Ionomycin Calcium Salt (Ionomycin, Solarbio, I8800) for 24 h. Concurrently, the low-, medium-, and high-dose CUR treatment groups received 1.5, 3, and 6 μM CUR, respectively, for 24 h. The experimental groups were designated as follows: blank control group (Ctrl), PMA and Ionomycin stimulation group (PMA + Iono), low-dose CUR treatment group (PI + CUR-L), medium-dose CUR treatment group (PI + CUR-M), and high-dose CUR treatment group (PI + CUR-H).

In subsequent functional validation experiments, cells underwent treatment with either TIGIT inhibitor (Aladdin, Ab169250) or NRP1 inhibitor (Aladdin, Ab183435) at a concentration of 5 μg/mL [22,23], following a 1 h pretreatment period. The medium-dose CUR treatment group was designated as the drug administration group. The experimental groups were defined as follows: blank control group (Ctrl); PMA and Ionomycin stimulation group (PMA + Iono); PMA + Iono with TIGIT inhibitor group (PI + TIGIT); PMA + Iono with NRP1 inhibitor group (PI + NRP1); PMA + Iono with both TIGIT and NRP1 inhibitors group (PI + TIGIT + NRP1); PI + TIGIT with medium-dose CUR treatment group (PIT + CUR-M); PI + NRP1 with medium-dose CUR treatment group (PIN + CUR-M); and PI + TIGIT + NRP1 with medium-dose CUR treatment group (PITN + CUR-M).

### 2.13. Enzyme-Linked Immunosorbent Assay (ELISA)

A piece of colon tissue weighing about 50 milligrams was collected for further examination and lysed using RIPA (RadioImmunoPrecipitation Assay) buffer (strong formulation; Biochem, Shenzhen, China) at a ratio of 1:10 (tissue weight to buffer volume). The samples were homogenized with an ultrasonic homogenizer for 10 min and subsequently incubated at 4 °C for one hour. Following the homogenisation process, the resulting mixtures were subjected to separation at 13,000 rpm and 4 °C for 30 min. The resulting supernatants were then retained for subsequent analytical procedures. Quantification of the total protein content in each sample relied on the application of the Ultra-Fast 5-Min BCA Protein Assay Kit (Suzhou Probelives Technology, P30008). Commercial ELISA facilitated the determination of IL-10, TGF-β1, IL-6, IL-17A, and TNF-α levels. Optical density (OD) at a wavelength of 450 nm was quantified using the Varioskan instrument, supplied by Thermo Fisher Scientific (Eugene, Oregon, USA). Quantification of cytokines was conducted by referencing standard curves established in accordance with the protocols provided by the ELISA kits.

### 2.14. Real-Time Quantitative PCR (RT-PCR)

Collect 20 mg of colon tissue or 5 × 10^6^ Jurkat cells and extract total RNA using the Tissue/Cell RNA Rapid Extraction Kit (Aidlab, RN68). Assess RNA quality through nucleic acid electrophoresis. Subsequently, normalize the cDNA to an equivalent concentration and perform reverse transcription using the Reverse Transcription Kit (Aidlab, PC7001) in accordance with the manufacturer’s protocol. The resulting cDNA samples were analyzed by real-time PCR employing the 2× Dual Sybr Green qPCR Mix (Aidlab, PC7001) on a Roche LightCycler 96 bioanalyzer (CFX96 Touch Real-Time PCR Detection System, Bio-Rad, Hercules, CA, USA). Primer sequences are detailed in Table 1. GAPDH served as the internal control gene, and relative target gene expression levels were quantified using the 2^(−ΔΔCt)^ method.

### 2.15. Western Blotting

Colon tissue extracts were standardized to a final concentration of 3.0 μg/μL, with Jurkat cells subjected to identical treatment conditions as the tissue samples. Each sample was normalised using the mean grayscale value of its corresponding β-actin. Standardized protein quantities from each sample were resolved by denaturing polyacrylamide gel electrophoresis (SDS-PAGE). The proteins were then transferred onto a polyvinylidene difluoride (PVDF) membrane using a Bio-Rad Western blot apparatus. The membranes were blocked with Western Blotting Blocking Buffer (Beyotime, P0023B-500mL) and incubated overnight at 4 °C with primary antibodies against β-actin (CST, 4970S, 1:1000), TIGIT (Proteintech, 83545-1-RR, 1:3000), and Neuropilin-1 (Proteintech, 84429-5-RR, 1:1000). Subsequent to incubation with the primary antibody, the membranes were treated with HRP-conjugated anti-rabbit IgG secondary antibody (CST, 7074S, 1:10,000) at ambient temperature for 1–2 h. Protein bands were identified utilizing the BioECL Ultra-Sensitive Chemiluminescent Detection Kit (Biochem, BKM-EL-200). Visualization was performed with the ChemiDoc MP imaging system (Bio-Rad, Hercules, CA, USA) and quantified using ImageJ software version 1.53k (Wayne Rasband and contributors, National Institutes of Health, Bethesda, MD, USA).

### 2.16. Immunofluorescence Staining

Mouse colon tissues were fixed, deparaffinized, and rehydrated according to the standard H&E staining protocol, followed by antigen retrieval to unmask antigenic epitopes. Subsequently, tissues were incubated with normal goat serum blocking solution at ambient temperature for 30 min to minimize nonspecific antibody binding. Primary antibodies against TIGIT (Proteintech, 83545-1-RR, 1:250) and Neuropilin-1 (Proteintech, 84429-5-RR, 1:250) were diluted in blocking solution and incubated with the tissues overnight at 4 °C.

On the following day, fluorescein isothiocyanate (FITC)-conjugated goat anti-rabbit IgG (H + L) secondary antibody (Proteintech, SA00003-2, 1:500), diluted in blocking solution, was applied under light-protected conditions and incubated under ambient temperature conditions for one hour. Cell nuclei were counterstained with DAPI staining solution (LEAGENE, 0305A25) at ambient temperature for 30 min in the absence of light. Finally, slides were mounted using an anti-fade mounting medium (BOSTER, AR1109). Staining results were examined, and images were acquired utilizing a fluorescence microscopy system (Leica, DM2000LED, Wetzlar, Germany) to assess the subcellular localization and abundance of the target proteins.

### 2.17. Statistical Analysis

The data analysis for this study was conducted utilizing GraphPad Prism version 10.5.0 software. The sample size (*n*) is determined based on preliminary experiments or estimations of biological variability. The arithmetic mean and standard error of the mean (SEM) were used to represent central tendency and variability for all quantifiable variables. Prior to performing one-way analysis of variance (ANOVA), it is essential to assess the assumptions of data normality and homogeneity of variance. Significant differences between groups were determined by ANOVA, which was followed by Tukey’s HSD test for multiple comparisons. Differences were deemed statistically significant when *p* < 0.05.

## 3. Results

### 3.1. CUR Effectively Alleviates DSS-Induced UC in Mice

To accurately replicate the pathophysiological features of human UC, we established a disease model using the classical DSS solution (Figure 1A) [24]. In this study, mice in the DSS group demonstrated a significant and progressive reduction of body mass from day 4 to day 21 compared to the Ctrl group (Figure 1B). Moreover, the DAI of DSS-induced mice was significantly higher compared to Ctrl group throughout days 1 to 21 (Figure 1C). Conversely, mice receiving CUR via gavage from day 7 to day 21 exhibited a notable increase in body weight, attenuation of DAI, and improved survival rates compared to the DSS group (Figure 1B–D). Importantly, on day 22, the DSS + CUR group showed significantly greater colon length, reduced colon and spleen weights, and decreased colon length/weight ratios, colon weight index, and spleen weight index relative to the DSS group (Figure 1E). The Ctrl + CUR group demonstrated statistical equivalence to Ctrl group. In summary, mice subjected to DSS developed characteristic clinical manifestations of ulcerative colitis without substantial improvement, whereas administration of CUR resulted in marked alleviation of clinical symptoms and disease progression.

Additionally, the results demonstrated intact mucosa with well-defined crypt architecture in both the Ctrl and Ctrl + CUR groups. In contrast, the colon of DSS group exhibited severe structural disruption, including massive inflammatory cell infiltration, crypt loss, and erosion of the mucosal architecture, which corresponded with significantly elevated histopathological damage scores (Figure 1F). Notably, the DSS + CUR group ameliorated these histopathological alterations, as evidenced by the preservation of crypt architecture and a reduction in inflammatory infiltration, resulting in significantly decreased histopathological scores (Figure 1F). Subsequently, we quantified the levels of key cytokines in colonic tissue. In comparison to the Ctrl and Ctrl + CUR groups, the levels of proinflammatory cytokines IL-6, IL-17A, and TNF-α were significantly increased in the colonic tissue of the DSS group (Figure 1G). Conversely, the production of the anti-inflammatory cytokines IL-10 and TGF-β1 was diminished (Figure 1G). Treatment with CUR significantly reversed these alterations by downregulating IL-6, IL-17A, and TNF-α expression, while enhancing the production of IL-10 and TGF-β1, thereby suggesting a reestablishment of immune homeostasis within the colon (Figure 1G). In conclusion, CUR significantly mitigates the symptoms of DSS-induced UC and demonstrates notable immunomodulatory properties.

### 3.2. CUR Restores T Cell and Memory T Cell Immune Homeostasis of Colitis Mice

**Figure 2:**
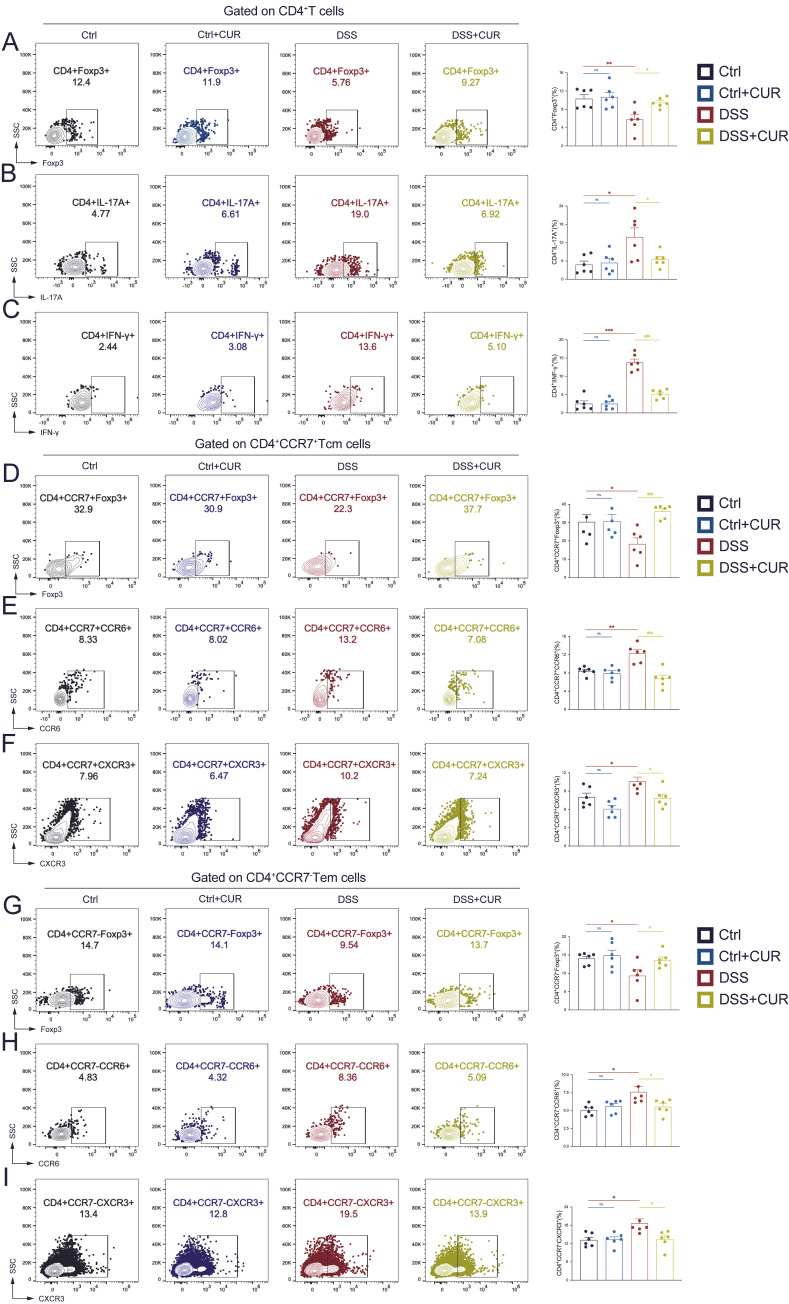
CUR Restores T cell and memory T cell Immune Homeostasis of Colitis Mice. Representative flow cytometric scatter plots accompanied by frequency distribution profiles of CD4^+^Foxp3^+^ Treg cells (**A**), CD4^+^IL-17A^+^ Th17 cells (**B**), CD4^+^INF-γ^+^ Th1 cells (**C**), CD4^+^CCR7^+^Foxp3^+^ Treg-Tcm cells (**D**), CD4^+^CCR7^+^CCR6^+^ Th17-Tcm cells (**E**), CD4^+^CCR7^+^CXCR3^+^ Th1-Tcm cells (**F**), CD4^+^CCR7^−^Foxp3^+^ Treg-Tem cells (**G**), CD4^+^CCR7^−^CCR6^+^ Th17-Tem cells (**H**) and CD4^+^CCR7^−^CXCR3^+^ Th1-Tem cells (**I**) in the four groups. The data are expressed as the mean ± SEM (*n* = 6). Significantly different (* *p* < 0.05, ** *p* < 0.01, *** p < 0.001) vs. the Ctrl group. Significantly different (^#^ *p* < 0.05, *^###^ p* < 0.001) vs. the DSS group.

Accumulating evidence suggests that CD4^+^ naïve T cells, CD4^+^ Tcm cells, and CD4^+^ Tem cells have a critical function in the development of autoimmune diseases, such as UC [25]. Compared to Ctrl group, the DSS-induced group exhibited a pronounced disruption in helper T cell homeostasis, as evidenced by a substantial decrease in CD4^+^Foxp3^+^ Treg cells (Figure 2A). Conversely, the proportions of pro-inflammatory CD4^+^IL-17A^+^ Th17 and CD4^+^IFN-γ^+^ Th1 cells were markedly elevated (Figure 2B,C). Within the DSS + CUR, the proportion of CD4^+^Foxp3^+^ Treg cells increased substantially (Figure 2A), while the percentages of CD4^+^IL-17A^+^ Th17 and CD4^+^IFN-γ^+^ Th1 cells were substantially decreased (Figure 2B,C). The Ctrl + CUR group, which did not receive DSS induction, exhibited no significant alterations in helper T cell populations compared to the Ctrl group.

Subsequently, we examined whether these alterations were also evident within memory T cell populations. In the Tcm subset: Compared to control mice, DSS-induced mice exhibited a marked decrease in CD4^+^CCR7^+^Foxp3^+^ Treg-Tcm cells, whereas CUR administration reversed this reduction (Figure 2D). Conversely, the frequencies of pathogenic CD4^+^CCR7^+^CCR6^+^ Th17-Tcm and CD4^+^CCR7^+^CXCR3^+^ Th1-Tcm cells were elevated in DSS-induced mice, and this increase was significantly attenuated by CUR treatment (Figure 2E,F). A similar pattern was observed in Tem subset: The proportion of CD4^+^CCR7^−^Foxp3^+^ Treg-Tem cells was markedly reduced in the DSS group (Figure 2G), whereas inflammatory CD4^+^CCR7^−^CCR6^+^ Th17-Tem and CD4^+^CCR7^−^CXCR3^+^ Th1-Tem cells were enriched (Figure 2H,I). In contrast to the DSS group, CUR-treated models exhibited a substantial decrease in the frequency of these inflammatory Tem subsets. This effect was accompanied by an increase in the prevalence of CD4^+^CCR7^−^Foxp3^+^ Treg-Tem cells (Figure 2G–I). Collectively, these findings demonstrate that CUR effectively restores immune homeostasis by rectifying imbalances across distinct T cell subsets, thereby underscoring its broad immunoregulatory potential in UC.

### 3.3. Network Pharmacology, Protein-Protein Docking, Molecular Docking, and Molecular Dynamics Simulations Were Identify to Predict the Key Targets of CUR in the Treatment of UC

**Figure 3:**
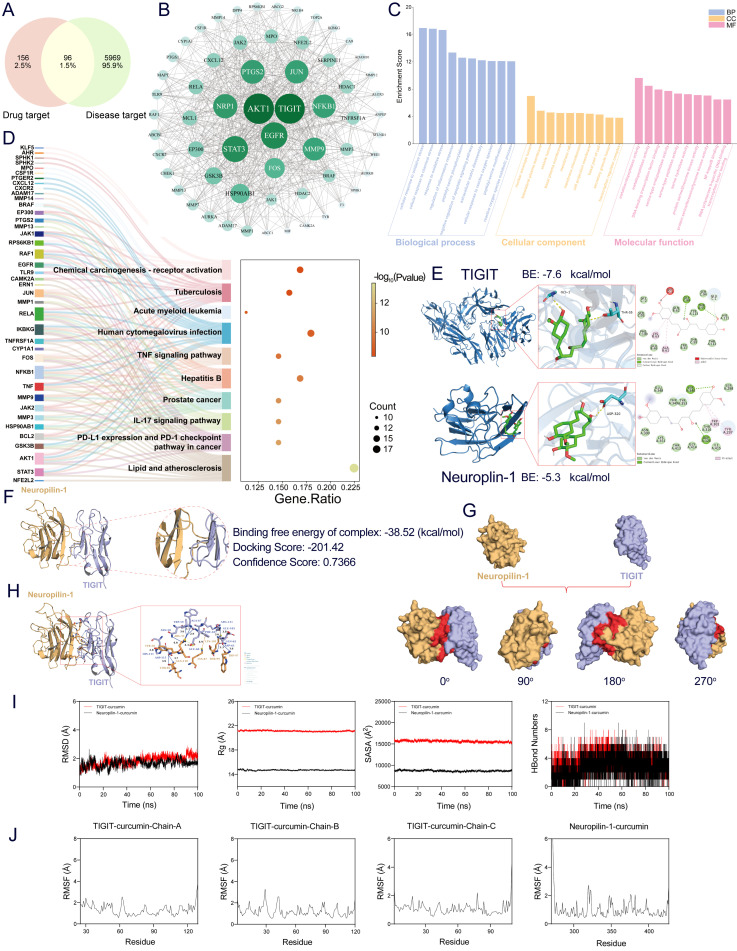
Network pharmacology, protein-protein docking, molecular docking, and molecular dynamics simulations were employed to identify the key targets of CUR in the treatment of UC. (**A**) Venn diagram of the intersection of the CUR target genes and UC target genes. (**B**) PPI Network Diagram of Core Targets for CUR and UC. (**C**) GO enrichment analysis: the top 10 BP, MF, and CC. (**D**) Bubble chart of the top 10 KEGG pathways. The larger the circle, the greater the number of target genes in the item. Additionally, color highlights the magnitude of the FDR: the redder the color, the smaller the value. (**E**) 2D and 3D Molecular Docking Analysis Diagrams of CUR Interacting with TIGIT and NRP1. (**F**) HDOCK server docking results: Neuropilin-1 (yellow) and TIGIT (purple). (**G**) Visualization of Surface-Based Alignment Results: Neuropilin-1 (yellow), TIGIT (purple), Protein-Protein Contact Area (red). (**H**) PLIP analysis reveals interactions between Neuropilin-1 (yellow) and TIGIT (purple). (**I**) CUR with TIGIT and NRP1 complexes molecular dynamics simulations: RMSD values over time; Rg values over time; SASA values over time; D. HBonds values over time; (**J**) RMSF values.

Venn diagrams were used to identify the active components of CUR obtained from PubChem. The candidate molecules targeted by CUR were predicted using the SwissTargetPrediction tool and the SEA database, resulting in 252 targets. Simultaneously, 6065 UC-associated targets were obtained from the GeneCards bioinformatics platform. Intersection analysis revealed 96 common targets shared between CUR and UC (Figure 3A). Subsequently, all component target data and intersecting target data were normalized. The VLOOKUP function was applied to match component targets based on the intersection of targets as selection criteria. These targets were subjected to network topology analysis to construct a “Chinese herbal medicine core component-target-disease” network diagram. This network visually delineates CUR’s target proteins, including TIGIT, AKT1, NRP1, STAT3, EGFR, MMP9, PTGS2, JUN, FOS, NF-κB1, among others (Figure 3B).

GO functional enrichment analysis demonstrated that the target molecules of CUR are significantly enriched in biological processes (BP), including oxidative stress response, regulation of inflammation, and reactive oxygen species metabolism. In terms of cellular components (CC), these targets localize to the cell membrane microdomain, secretory granule lumen, and transcription complex. Molecular function (MF) enrichment indicated involvement in metallopeptidase activity, serine/threonine kinase activity, and transcription factor binding, suggesting that CUR may exert therapeutic effects through multi-level regulation of oxidative damage, inflammatory responses, and gene expression pathways (Figure 3C). Notably, KEGG pathway analysis demonstrated that CUR’s potential therapeutic targets for ulcerative colitis are significantly enriched in pathways such as TNF, IL-17, and PD-L1/PD-1 checkpoint pathways related to cancer immunity, lipid metabolism and atherosclerosis, as well as infection- and immunity-associated pathways including tuberculosis, human cytomegalovirus infection, hepatitis B, and acute myeloid leukemia (Figure 3D). Although the analysis indicates a complex multi-target, multi-pathway network, the enriched pathways (e.g., IL-17 signaling, PD-1 checkpoint signaling) and key molecules (e.g., AKT1, STAT3) correspond closely with the established immune functions of TIGIT and NRP1. As a critical T-cell immune checkpoint receptor, TIGIT’s interaction with its co-receptor NRP1 is essential for suppressing T-cell overactivation and preserving immune system equilibrium. These results indicate that CUR may modulate the TIGIT/NRP1 axis, thereby suppressing intestinal inflammation and exerting therapeutic effects against UC.

Molecular docking simulations predicted that CUR forms strong attachments to both TIGIT and NRP1. These binding events yielded free energy scores of −7.6 kcal/mol for TIGIT and −5.3 kcal/mol for NRP1. These interactions involve the formation of stable hydrogen bonds and hydrophobic contacts within their respective active sites (Figure 3E). Protein-protein docking between TIGIT and Neuropilin-1 indicated a high likelihood of interaction, as evidenced by a docking score of −201.42 and a confidence score of 0.7366 (Figure 3F). Surface modeling visualization of the binding interface (Figure 3G) and detailed interaction analysis using PLIP (Figure 3H) revealed critical noncovalent interactions, including multiple hydrogen bonds and salt bridges formed between TIGIT residue ASP-115 and Neuropilin-1 residues THR-85 and GLN-110, thereby confirming the formation of a stable and specific protein-protein complex.

Following the initial analysis, a 100 ns MD simulation was performed to assess the equilibrium state and dynamic characteristics inherent to the CUR–target protein complexes. The RMSD values for the TIGIT-CUR and Neuropilin-1-CUR complexes stabilized within acceptable thresholds, remaining below 2 Å and 1.6 Å, respectively, following initial equilibration, which indicates robust structural stability (Figure 3I, left panel). Additional parameters, including the Rg, SASA, and hydrogen bond count, remained consistent throughout the simulation, suggesting that ligand binding did not induce significant structural perturbations (Figure 3I). Furthermore, low RMSF values, predominantly below 2 Å, confirmed the limited flexibility and high stability of residue regions in both complexes (Figure 3J). Collectively, these molecular dynamics results corroborate the molecular docking data, predicting that CUR establishes stable and favorable interactions with both TIGIT and NRP1.

### 3.4. CUR Regulate T Cell Immune Homeostasis via Intervening TIGIT Expression

**Figure 4:**
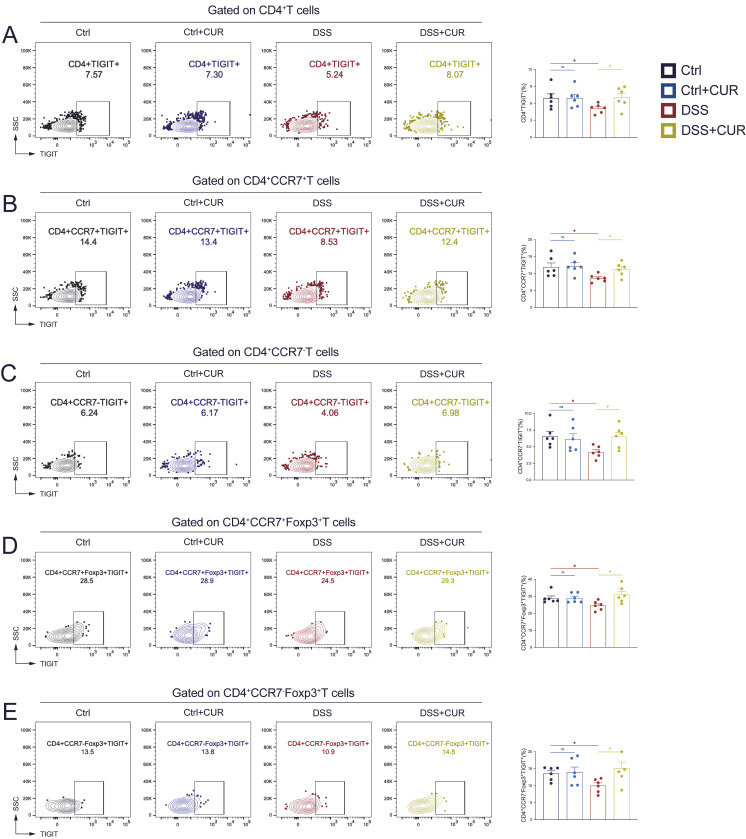
CUR regulates T cell immune homeostasis via intervening TIGIT expression. Representative flow cytometry plots and frequencies of CD4^+^TIGIT^+^ T cells (**A**), CD4^+^CCR7^+^TIGIT^+^ Tcm cells (**B**), CD4^+^CCR7^−^TIGIT^+^ Tem cells (**C**), CD4^+^CCR7^+^Foxp3^+^TIGIT^+^ cells (**D**), and CD4^+^CCR7^−^Foxp3^+^TIGIT^+^ cells (**E**) in the four groups. The data are expressed as the mean ± SEM (*n* = 6). Significantly different (* *p* < 0.05) vs. the Ctrl group. Significantly different (^#^ *p* < 0.05) vs. the DSS group.

Previous experimental results demonstrated that CUR regulates the balance of T cell subsets to maintain immune homeostasis. In subsequent experiments, we analyzed TIGIT expression on different CD4^+^ T cell subsets. In the DSS group, the frequency of TIGIT^+^ cells within total CD4^+^ T cells was significantly reduced, indicating impaired immunoregulatory function (Figure 4A). Following CUR treatment, the percentage of CD4^+^TIGIT^+^ T cells markedly increased, suggesting a restorative effect (Figure 4A). In CD4^+^CCR7^+^ Tcm cells, the proportion of TIGIT^+^ decreased in the DSS group but increased after CUR treatment (Figure 4B). Similarly, in effector memory-like CD4^+^CCR7^−^ T cells, DSS induction caused a significant decline in TIGIT expression, which was substantially reversed by CUR administration (Figure 4C). Interestingly, CUR also significantly enhanced TIGIT expression on Treg cells. In the CD4^+^CCR7^+^Foxp3^+^ T cell subset (central Tregs), DSS induction resulted in a significant reduction in TIGIT^+^ cells compared to controls, which was effectively restored by CUR treatment (Figure 4D). A similar trend was observed in CD4^+^CCR7^−^Foxp3^+^ T cells (effector Tregs), where CUR reversed the DSS-induced reduction in TIGIT^+^ frequency (Figure 4E). These findings indicate that CUR specifically upregulates TIGIT expression on CD4^+^ T cells, memory T cells, and memory Treg cells.

### 3.5. CUR Inhibits the Gene and Protein Expression of TIGIT/NRP1 in Colitis Mice

**Figure 5:**
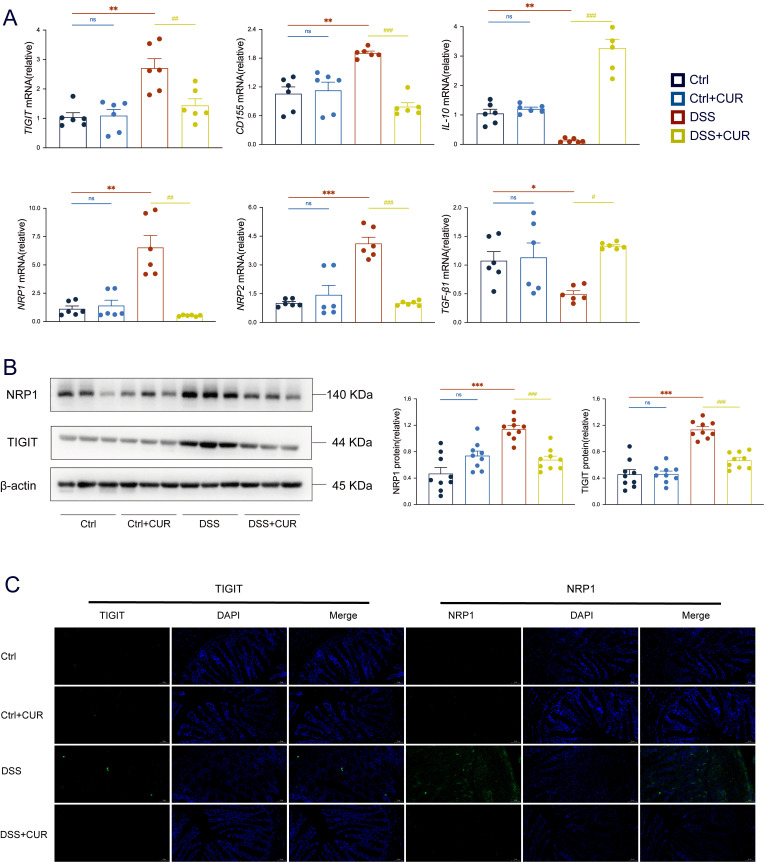
CUR inhibits the gene and protein expression of TIGIT/NRP1 signaling molecules in colitis mice. (**A**) Relative mRNA expression of TIGIT, CD155, IL-10, NRP1, NRP2, and TGF-β1 in colonic tissues. (**B**) The expression and quantitative evaluations of TIGIT and NRP1 in colonic tissues were analysed by western blotting, with GAPDH serving as a reference for total proteins. (**C**) Immunofluorescence expression of TIGIT and NRP1 in colon tissues of various groups, magnification 200 ×, Bar = 50 μm. The data are expressed as the mean ± SEM (*n* = 6–9). Significantly different (* *p* < 0.05, ** *p* < 0.01, *** *p* < 0.001) vs. the Ctrl group. Significantly different (^#^ *p* < 0.05, ^##^ *p* < 0.01, ^###^ *p* < 0.001) vs. the DSS group.

RT-qPCR results showed that DSS-induced inflammation led to an upregulation of mRNA expression for TIGIT, its ligand CD155, and the co-receptors NRP1 and NRP2, whereas CUR treatment significantly downregulated their transcriptional levels. Additionally, CUR increased the mRNA expression of IL-10 and TGF-β1, both essential for maintaining intestinal immune homeostasis (Figure 5A). Consistent with the RT-qPCR findings, Western blot analysis demonstrated significantly elevated protein levels of TIGIT and NRP1 in the DSS group, suggesting a potential compensatory immune regulatory response. CUR treatment markedly reduced the expression of these proteins, indicating its inhibitory effect on TIGIT and NRP1 (Figure 5B). Immunofluorescence staining further confirmed these protein expression patterns and provided spatial localization information. Strong fluorescent signals for TIGIT and NRP1 were observed in DSS-induced mice, which were significantly diminished following CUR treatment (Figure 5C). Collectively, these results suggest that CUR ameliorates experimental colitis by targeting the TIGIT/NRP1 axis, modulating gene and protein expression, and promoting an anti-inflammatory cytokine environment.

### 3.6. CUR Inhibits the Activation and Expression of TIGIT/NRP1 Signaling Molecules In Vitro

**Figure 6:**
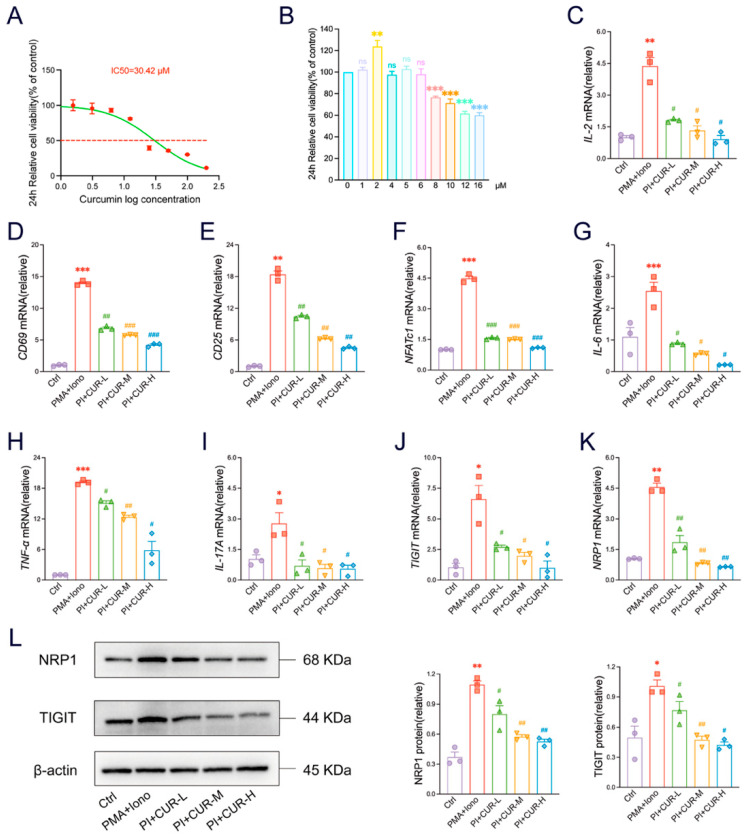
CUR inhibits the activation and expression of TIGIT/NRP1 signaling molecules *in* *vitro*. (**A**) The 24 h IC_50_ curve of CUR on Jurkat cells. The red dashed line represents the standard line corresponding to the IC_50_. (**B**) The optimal dose of CUR for Jurkat cells at 24 h. (**C**–**K**) Transcriptional abundance in Jurkat cells was quantified for key factors: IL-2, CD69, CD25, NFATc1, IL-6, TNF-α, IL-17A, TIGIT, and NRP1. (**L**) The expression and quantitative evaluations of TIGIT and NRP1 in Jurkat cells were analysed by Western blotting, with β-actin serving as a reference for total proteins. The data are expressed as the mean ± SEM (*n* = 3). Significantly different (* *p* < 0.05, ** *p* < 0.01, *** *p* < 0.001) vs. the Ctrl group. Significantly different (^#^ *p* < 0.05, ^##^ *p* < 0.01, ^###^ *p* < 0.001) vs. the DSS group.

To investigate the immediate immunomodulatory impacts of CUR on T cells, we used the Jurkat Clone E6-1 cell line. First, the 24 h IC_50_ value of CUR was determined to be 30.42 μM. Based on time- and dose-dependent experiments, we selected three non-cytotoxic concentrations-1.5, 3, and 6 μM-for subsequent evaluation of immunomodulatory effects (Figure 6A,B). In PMA and ionomycin-activated Jurkat cells, CUR treatment significantly suppressed mRNA expression of key T-cell activation markers. We observed dose-dependent decreases in IL-2, CD69, CD25, and the transcription factor NFATc1 (Figure 6C–F), indicating inhibited T-cell activation and proliferation. Furthermore, CUR significantly decreased the levels of IL-6, TNF-α, and IL-17A (Figure 6G–I), suggesting broad anti-inflammatory effects. Notably, CUR also modulated the expression of immune checkpoint-associated molecules, significantly downregulating mRNA levels of TIGIT and its co-receptor NRP1 (Figure 6J,K). Western blot analysis further confirmed this downregulation, showing reduced protein expression of both TIGIT and NRP1 following CUR treatment (Figure 6L). These lab findings indicate that CUR can block T cell activation, decrease the production of pro-inflammatory cytokines, and lower the expression of TIGIT and NRP1 genes and proteins in T cells.

### 3.7. CUR Inhibits the Inflammatory Response of T Cells via Blocking the Activation of TIGIT/NRP1 Expression In Vitro

**Figure 7:**
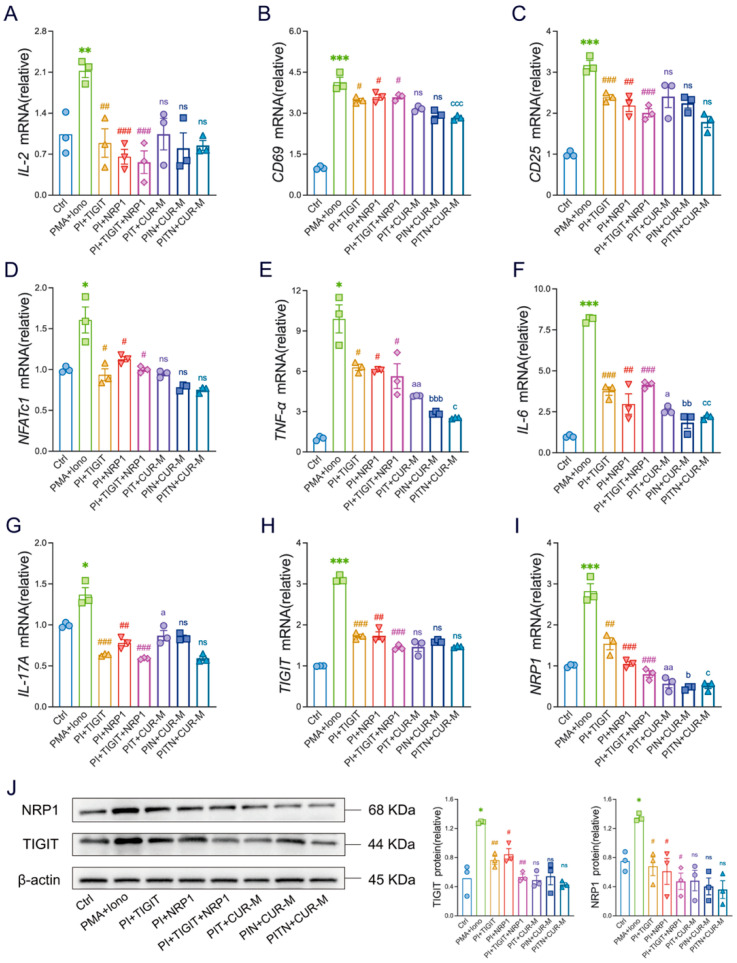
CUR directly inhibits the inflammatory response of T cells via blocking the activation of TIGIT/NRP1 expression *in* *vitro*. (**A**–**I**) Transcriptional abundance in Jurkat cells was quantified for key factors: IL-2, CD69, CD25, NFATc1, IL-6, TNF-α, IL-17A, TIGIT, NRP1. (**J**) The expression and quantitative evaluations of TIGIT and NRP1 in Jurkat cells were analysed by Western blotting, with β-actin serving as a reference for total protein. The data are expressed as the mean ± SEM (*n* = 3). Significantly different (* *p* < 0.05, ** *p* < 0.01, *** *p* < 0.001) vs. the Ctrl group. Significantly different (^#^ *p* < 0.05, ^##^ *p* < 0.01, *^###^ p* < 0.001) vs. the PMA + Iono group. Significantly different (^a^ *p* < 0.05, ^aa^ *p* < 0.01) vs. the PI + TIGIT group. Significantly different (^b^ *p* < 0.05, ^bb^ *p* < 0.01, ^bbb^ *p* < 0.001) vs. the PI + NRP1 group. Significantly different (^c^ *p* < 0.05, ^cc^ *p* < 0.01, ^ccc^ *p* < 0.001) vs. the PI + TIGIT + NRP1 group.

Subsequently, we performed functional validation experiments. These experiments involved treating Jurkat cells with a TIGIT inhibitor, an NRP1 inhibitor, and a combination of both inhibitors, followed by quantification of mRNA expression levels of key T cell activation markers, including IL-2, CD69, CD25, and NFATc1. Compared to the activation group stimulated solely with PMA and ionomycin, all inhibitor-treated groups demonstrated significant suppression of these activation markers (Figure 7A–D). Furthermore, co-treatment of each inhibitor group with CUR further reduced mRNA levels of IL-2, CD69, CD25, and NFATc1 relative to the respective inhibitor-only groups; however, the findings were not statistically distinct between the co-treated groups and their corresponding inhibitor-only groups (Figure 7A–D).

Concerning pro-inflammatory cytokines, all inhibitor-treated groups, as well as those co-cultured with both inhibitors and CUR, exhibited a significant decrease in the expression of IL-6, TNF-α, and IL-17A (Figure 7E–G). Notably, the reductions were more pronounced in the co-culture groups receiving both inhibitors and CUR. These findings indicate that, in the presence of TIGIT/NRP1 inhibitors, the inhibitory effect of CUR was effectively antagonized, as evidenced by the absence of notable differences between the inhibitor-only groups. Those treated with both inhibitors and CUR. This suggests that TIGIT/NRP1 serves as a critical positive regulatory mechanism mediating Jurkat cell activation; consequently, blocking them effectively suppresses T cell activation and diminishes the production of associated inflammatory cytokines.

Furthermore, we measured the mRNA levels of TIGIT and its co-receptor NRP1 quantitatively. The findings demonstrated that treatment with the TIGIT inhibitor, NRP1 inhibitor, and their combination significantly downregulated the expression of the corresponding mRNAs (Figure 7H,I). This inhibitory effect was further enhanced following co-culture with CUR. These observations were corroborated by Western blot analysis (Figure 7J). In summary, although the effect of curcumin was largely attenuated, its mechanism of action appears to be more complex than mere receptor blockade. CUR may not only inhibit the activity of TIGIT and NRP1 but also decrease their protein expression levels by modulating transcriptional processes or post-translational modifications, thereby complementing and potentiating the functional blockade exerted by the inhibitors.

## 4. Discussion

Overall, our findings indicate that CUR significantly ameliorates typical clinical and pathological symptoms in DSS-induced mice, including weight loss, elevated DAI, shortened colon length, and histological damage. These improvements were highly correlated with restoration of the intestinal immune microenvironment, manifested by significant reductions in proinflammatory cytokines and increases in anti-inflammatory cytokines. These results indicate that CUR effectively mitigates the clinical and pathological manifestations of UC by modulating the intestinal immune microenvironment and suppressing excessive inflammatory responses. Our flow cytometric analysis revealed that CUR effectively corrected the imbalance of key T cell subsets in the UC model. This restoration of T cell balance extended beyond effector T cells to memory T cell subsets (Tcm and Tem), providing compelling evidence for CUR’s role in maintaining long-term immune homeostasis. Thus, the therapeutic effects of CUR are both multifaceted and substantial; its immunomodulatory influence encompasses not only short-term Tcm cells but also Tem cell populations that mediate long-term immune responses. By modulating memory T cells, CUR may intervene in the chronic progression and relapse risk associated with the disease.

During the progression of UC, TIGIT exhibits a dual role, contributing both to immune suppression and, under certain conditions, potentially to pro-inflammatory responses. Research has shown that subsequent to achieving clinical remission in UC patients, the population of Treg cells expressing TIGIT (CD226^–^TIGIT^+^Foxp3^+^) is restored, suggesting that TIGIT is integral to Treg-mediated immune suppression and disease resolution [26]. In contrast to its immunosuppressive function, research using murine models of colitis has revealed that the absence of TIGIT may alleviate intestinal inflammation and tissue damage [27]. Unlike TIGIT’s dual functionality, NRP1 is predominantly associated with pro-inflammatory roles. It has been shown that NRP1 modulates and enhances the capacity of ILC3s to produce the pro-inflammatory cytokine IL-17A, thereby directly promoting and exacerbating colitis [18]. Employing network pharmacology, protein docking, and MD simulations, we predicted a pivotal role for TIGIT/NRP1 in the therapeutic effects of CUR in UC. These results solely predict the feasibility and stability of the interaction between CUR and TIGIT/NRP1. However, further experimental validation remains necessary.

Moreover, our research findings indicate that the DSS group exhibited a marked increase in the overall mRNA and protein expression levels of TIGIT and NRP1. Conversely, following CUR treatment, these levels in inflamed colonic tissue were significantly reduced relative to the DSS-induced mice. Furthermore, CUR regulates TIGIT expression within T cell subsets. In the DSS group, the proportion of TIGIT^+^ cells among total CD4^+^ T cells, within functional Treg subsets (Treg-Tcm and Treg-Tem), was significantly decreased, indicating impaired immune tolerance under disease conditions. Notably, CUR treatment reversed this DSS-induced reduction, significantly restoring the frequency of TIGIT^+^ cells within the memory Treg subsets. A diminished proportion of TIGIT^+^ Tregs is recognized as a hallmark of autoimmune disease pathogenesis [28]. We recognized that TIGIT expression is closely associated not only with the stability of Tregs but also with the transient activation and exhaustion states of effector and memory T cells. This apparent paradox may be elucidated by the recruitment of immune cells to sites of intestinal inflammation following disease onset, where pathogenic immune cells extensively infiltrate and provoke an overactivation of the immune system. Consequently, this results in the sustained upregulation of TIGIT and NRP1. Due to immune dysregulation, mice treated with DSS are unable to adequately regulate this aberrant response. CUR, known for its potent anti-inflammatory properties, has been shown to effectively suppress these pathogenic immune cells, thereby reducing the expression of TIGIT and NRP1 and contributing to the restoration of intestinal immune homeostasis. The observed decrease in the frequency of TIGIT-positive T cells across all CD4^+^ subsets may indicate a broader systemic immune deficiency. In the chronic ulcerative colitis model, during the late inflammatory phase, DSS-treated mice may experience systemic T cell functional exhaustion or increased apoptosis, resulting in a widespread loss of TIGIT expression across subsets and impaired peripheral immune tolerance.

*In vitro* studies have demonstrated that CUR modulates TIGIT and NRP1, which constitutes a critical mechanism for the inhibition of T cell activation. At non-cytotoxic concentrations, CUR dose-dependently decreased both mRNA and protein expression levels of TIGIT and NRP1. Additionally, CUR inhibited the expression of T cell activation markers, including IL-2, CD69, and CD25, as well as the transcription factor NFATc1, and it also reduced the levels of IL-6, TNF-α, and IL-17A. This finding is consistent with the observed upregulation of TIGIT and NRP1 in the colonic tissues of DSS-treated mice. We propose that, within the simplified and highly activated Jurkat cell model, the elevated expression of TIGIT and NRP1 results from the activation of specific inflammatory signaling pathways. Moreover, CUR appears to inhibit these pathways, thereby suppressing the expression of transcription factors associated with T cell activation, as well as TIGIT and NRP1.

Subsequently, functional validation experiments were conducted to determine whether the mechanism of CUR is entirely dependent on TIGIT/NRP1. The results indicate that the application of TIGIT or NRP1 inhibitors effectively impedes T cell activation. When CUR is administered in conjunction with these inhibitors, there is a more pronounced suppression of T cell activation markers (IL-2, CD69, CD25, NFATc1) and pro-inflammatory cytokines (IL-6, TNF-α, IL-17A), although this enhancement does not attain statistical significance. These findings suggest that TIGIT/NRP1 constitutes the principal pathway through which CUR modulates T cell activation. Furthermore, co-culturing with both the inhibitors and CUR appears to produce a synergistic or additive effect. This synergy indicates that CUR’s mechanism of action predominantly relies on TIGIT/NRP1, while it is also likely to concurrently inhibit other critical parallel signalling pathways, or directly inhibit NFATc1, all of which contribute to T cell activation and cytokine production [29]. By modulating TIGIT/NRP1 alongside the suppression of these fundamental T cell signalling pathways, CUR achieves enhanced immunosuppressive effects, thereby confirming its role as a multi-target therapeutic agent. Utilizing inhibitors of TIGIT and NRP1, we demonstrated that this inhibitory effect primarily functions by impeding the transcriptional and translational activities of TIGIT and NRP1, rather than solely modulating the expression of inflammation-related cytokines. Future research will focus on elucidating the upstream and downstream signaling pathways of TIGIT and NRP1 to clarify the precise mechanisms by which CUR regulates these molecules.

Previous studies have predominantly concentrated on the suppressive impacts of curcumin on key inflammatory pathways, such as NF-κB and MAPK [30]. However, there is a paucity of research regarding its regulatory effects on immune checkpoint molecules. This study elucidates the connection between CUR well-established broad-spectrum anti-inflammatory properties and the T cell-specific TIGIT/NRP1 regulatory axis, thereby significantly enhancing the understanding of CUR’s pharmacological mechanisms. It provides novel theoretical foundations and practical implications for the treatment of UC. The findings extend beyond the conventional perception of CUR as solely a broad-spectrum antioxidant or NF-κB inhibitor, demonstrating that natural compounds can also mediate targeted immune modulation, a function traditionally attributed to highly specific biologic agents. Considering the involvement of TIGIT and NRP1 in T cell exhaustion and chronic inflammation, CUR’s modulation of this axis offers new perspectives for therapeutic intervention in other chronic autoimmune or inflammatory diseases characterized by T cell dysfunction. CUR not only targets the critical TIGIT/NRP1 regulatory axis but also suppresses downstream effector pathways, including cytokine production and NFATc1 activity. This integrated mechanism comprehensively accounts for CUR’s potent and multifaceted efficacy observed in preclinical models.

Regarding mechanistic details, although MD simulations have predicted its stability, higher-resolution structural biology techniques, such as cryo-electron microscopy or X-ray crystallography, are required to precisely delineate the binding interfaces of CUR with TIGIT and NRP1. This is essential to ascertain whether CUR functions as an allosteric modulator or directly inhibits ligand-binding domains, for example, the TIGIT-CD155 interaction. Methodologically, this study utilized immortalized Jurkat cell lines. While these models are valuable for mechanistic investigations, validation in more physiologically relevant systems, including primary human intestinal lamina propria T cells, is necessary to confirm the applicability of the TIGIT/NRP1 mechanism within the human intestinal microenvironment. The results of this research offer a novel perspective on the complex mechanisms underlying CUR treatment for UC. Previous research has primarily focused on CUR’s inhibitory effects on core inflammatory signaling pathways, including NF-κB and MAPK [31,32], with limited reports on its regulatory role in immune checkpoint molecules. By linking CUR’s known broad-spectrum anti-inflammatory properties to the specific T cell regulatory axis TIGIT/NRP1, this study successfully fills this knowledge gap.

## 5. Conclusions

In summary, this study not only confirms CUR’s significant therapeutic efficacy in UC but also systematically elucidates for the first time its molecular mechanism of restoring T-cell immune balance by targeting and regulating the TIGIT/NRP1 immune checkpoint axis. This discovery provides crucial theoretical foundations and research directions for developing novel CUR-based therapeutic strategies for UC, particularly combination regimens integrating TIGIT/NRP1. Certainly, the present study has several limitations. Notably, TIGIT/NRP1 knockout or knock-in mouse models were not employed to induce DSS-induced colitis, and the bioavailability of CUR in colonic mucosa and lymphoid tissues was not assessed. Crucially, the bioavailability of CUR remains a significant barrier to clinical translation, necessitating further pharmacokinetic studies and optimization of its formulation. It is possible that the active constituents of CUR, produced through intestinal flora catabolism, exert significant effects in regulating T cell immune homeostasis. In future research, we plan to utilize both global and conditional knockout models of the TIGIT/NRP1 genes in mice, including specific T cell populations, to induce colitis and develop cellular models of UC. Such investigations hold considerable scientific interest and promise. Additionally, we are developing enhanced delivery systems for CUR to realize the full therapeutic potential of this promising compound.

## Figures and Tables

**Table 1:** Primer sequences.

Primer	Forward (5′-3′)	Reverse (5′-3′)
*GAPDH (mouse)*	CATCACTGCCACCCAGAAGACTG	ATGCCAGTGAGCTTCCCGTTCAG
*TIGIT (mouse)*	CCACAGCAGGCACGATAGATA	CATGCCACCCCAGGTCAAC
*NRP1 (mouse)*	CAGGGTTTTCCATCCGCTATG	ACTCCAGTAGGTGCTGTATAGTT
*CD155 (mouse)*	GGCCCTCGAATGTGAATGGAA	CGTGCAGGTGATGTTCTTGC
*NRP2 (mouse)*	GCTGGCTACATCACTTCCCC	GGGCGTAGACAATCCACTCA
*IL-10 (mouse)*	AGCCTTATCGGAAATGATCCAGT	GGCCTTGTAGACACCTTGGT
*TGF-β1 (mouse)*	CTTCAATACGTCAGACATTCGGG	GTAACGCCAGGAATTGTTGCTA
*TIGIT (human)*	TGGTGGTCATCTGCACAGCAGT	TTTCTCCTGAGGTCACCTTCCAC
*NRP1 (human)*	AACAACGGCTCGGACTGGAAGA	GGTAGATCCTGATGAATCGCGTG
*GAPDH (human)*	GTCTCCTCTGACTTCAACAGCG	ACCACCCTGTTGCTGTAGCCAA
*IL-2 (human)*	AGAACTCAAACCTCTGGAGGAAG	GCTGTCTCATCAGCATATTCACAC
*IL-6 (human)*	AGACAGCCACTCACCTCTTCAG	TTCTGCCAGTGCCTCTTTGCTG
*TNF-α (human)*	CTCTTCTGCCTGCTGCACTTTG	ATGGGCTACAGGCTTGTCACTC
*NFATc1 (human)*	CACCAAAGTCCTGGAGATCCCA	TTCTTCCTCCCGATGTCCGTCT
*CD69 (human)*	GCTGGACTTCAGCCCAAAATGC	AGTCCAACCCAGTGTTCCTCTC
*CD25 (human)*	GAGACTTCCTGCCTCGTCACAA	GATCAGCAGGAAAACACAGCCG

## Data Availability

The original contributions presented in this study are included in the article. Further inquiries can be directed to the corresponding authors.
